# LncRNA HOTAIR regulates the PI3K/AKT pathway via the miR‐126‐3p/PIK3R2 axis to participate in synovial angiogenesis in rheumatoid arthritis

**DOI:** 10.1002/iid3.1064

**Published:** 2023-10-27

**Authors:** Feifei Liu, Yuan Wang, Dan Huang, Yanqiu Sun

**Affiliations:** ^1^ Graduate School Anhui University of Traditional Chinese Medicine Hefei Anhui China; ^2^ Department of Rheumatology The First Affiliated Hospital of Anhui University of Traditional Chinese Medicine Hefei Anhui China

**Keywords:** angiogenesis, LncRNA HOTAIR, PI3K/AKT, PIK3R2, rheumatoid arthritis

## Abstract

**Background:**

The abnormal expression of long noncoding RNA (LncRNA) HOTAIR has been associated with synovial angiogenesis in rheumatoid arthritis (RA). The aim of this study is to investigate whether LncRNA HOTAIR plays a role in synovial angiogenesis in RA by regulating the phosphoinositide 3‐kinase/protein kinase B (PI3K/AKT) pathway through the miR‐126‐3p/PIK3R2 axis.

**Methods:**

In this study, we conducted in vitro experiments by designing overexpression plasmids and small interfering RNAs targeting LncRNA HOTAIR and then transfected them into rheumatoid arthritis fibroblast‐like synoviocytes (RA‐FLS). We then co‐cultured the RA‐FLS with human umbilical vein endothelial cells (HUVEC) to establish a RA‐FLS‐induced HUVEC model. We investigated the effects of LncRNA HOTAIR on the proliferation, migration, lumen forming ability of HUVEC, as well as the expression of synovial endothelial cell markers, angiogenic factors, and the PI3K/AKT pathway. To validate the interactions between LncRNA HOTAIR, miR‐126‐3p, and PIK3R2, we used bioinformatics and luciferase reporter experiments. We also employed real‐time fluorescence quantitative, Western blotanalysis, and immunofluorescence techniques to analyze the target genes and proteins.

**Results:**

The expression of LncRNA HOTAIR was upregulated in HUVEC induced by RA‐FLS. The overexpression of LncRNA HOTAIR significantly increased the expression of vascular endothelial growth factor, basic fibroblast growth factor, CD34, and CD105 in HUVEC, promoting their proliferation, migration, and lumen formation. At the same time, the overexpression of LncRNA HOTAIR inhibited the expression of miR‐126‐3p, promoted the expression of PIK3R2, activated the PI3K/AKT pathway, and promoted the expression of PI3K, AKT and phosphorylated‐AKT, while the silence of LncRNA HOTAIR reversed these expressions. Bioinformatics and double luciferase reporter gene experiments confirmed the targeting relationship among LncRNA HOTAIR, miR‐126‐3p, and PIK3R2. Finally, the rescue experiments showed that PI3K agonists could reverse the inhibitory effect of silent LncRNA HOTAIR on HUVEC.

**Conclusion:**

LncRNA HOTAIR has the potential to activate the PI3K/AKT pathway, likely through the regulatory axis involving miR‐126‐3p/PIK3R2, consequently contributing to synovial angiogenesis in RA.

## Graphical abstract

Our study sheds light on the potential role of neovascularization in the pathogenesis of rheumatoid arthritis (RA). The long‐chain noncoding RNA HOTAIR/miR‐126‐3p/PIK3R2 signaling axis may contribute to RA neovascularization and provide novel insights and potential targets for therapeutic intervention in RA.

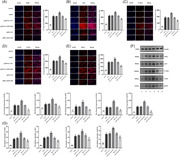

## 1 INTRODUCTION

Rheumatoid arthritis (RA) is a complex autoimmune disease that is difficult to treat and has a high rate of recurrence.^1^ It primarily involves synovitis and vasculitis, with an unclear understanding of its pathogenesis.^2^ Clinical manifestations associated with RA include joint swelling, pain, morning stiffness, and irreversible deformity changes.^3^ Previous studies have reported that angiogenesis promotes the inflammatory response and accelerates synovial hyperplasia leading to bone destruction.^4^, ^5^ Angiogenesis is a complicated process that allows the growth and repair of tissues by forming new blood vessels from pre‐existing ones. It involves the activation, proliferation, migration, and differentiation of endothelial cells in response to various activating factors. Endothelial cells gradually migrate toward the basement membrane and form new blood vessels through budding in a pattern of tissue growth. Angiogenesis is crucial for various physiological processes, including embryonic development, wound healing, and tissue regeneration, as well as pathophysiological conditions like cancer and inflammation.^6^ Fibroblast‐like synoviocytes (FLS) are the primary effect cells sustaining joint inflammation, angiogenesis, and cartilage destruction.^7^ During RA activity, FLS produces a multitude of angiogenic factors, including vascular endothelial growth factor (VEGF) and basic fibroblast growth factor (bFGF), which pathologically alter endothelial cell activity and promote joint angiogenesis. This exacerbates RA severity by contributing to synovial hyperplasia and inflammation.^8^ Therefore, regulating angiogenesis induced by FLS is a potential strategy for the treatment of RA.

Long noncoding RNAs (LncRNAs) participate in the translation modification of genes and regulation of the expression of biological molecules such as endothelial cells, inflammatory factors, and messenger RNAs.^9^, ^10^ There is an abnormal expression of LncRNA in blood and synovial tissue of RA patients,^11^ which is an important potential target for the treatment of RA.^12^, ^13^ LncRNAs play a crucial role in regulating synovial angiogenesis and are actively involved in the inflammatory process of RA.^14^ Previous research has shown that the expression of the LncRNA HOTAIR is noticeably increased in individuals with RA incontrast to healthy controls.^15^ In addition, studies have shown that transfecting RA‐FLS with small interfering RNA (siRNA) targeting lncRNA HOTAIR leads to decreased proliferation, invasion, and migration abilities compared to overexpressed groups.^16^ The activation of the phosphoinositide 3‐kinase/protein kinase B (PI3K/AKT) signaling pathway can significantly enhance the proliferation, migration, and tubule formation of human umbilical vein endothelial cells (HUVECs). Moreover, this pathway exhibits potent antiangiogenic effects both in vivo and in vitro.^17^ As a regulatory subunit of class IA PI3K enzymes, the PIK3R2 protein is activated by tyrosine kinase receptors, thereby mediating the PI3K/AKT pathway.^18^ Li et al. identified that miR‐126 exerts regulatory control over the PI3K/AKT signaling pathway by targeting and inhibiting PIK3R2, including angiogenesis, cell proliferation, differentiation, and migration.^19^, ^20^ During our bioinformatics analysis, we discovered a potential binding site between LncRNA HOTAIR and miR‐126‐3p. It is currently unclear whether HOTAIR plays a role in modulating the PI3K/AKT pathway through the miR‐126‐3p/PIK3R2 axis to contribute to synovial angiogenesis in RA. Further research is needed to investigate the potential involvement of HOTAIR in this regulatory mechanism.

Our study aimed to assess the impact of HOTAIR on various aspects of HUVEC activity, such as proliferation, migration, tube formation, and expression of vascular markers, under the influence of RA‐FLS stimulation. We specifically investigated the role of the LncRNA HOTAIR/miR‐126‐3p/PIK3R2 axis in regulating angiogenic factors and the PI3K/AKT pathway. Through our research, we aimed to provide insights into the mechanisms involved in synovial angiogenesis mediated by RA and identify potential therapeutic targets.

## 2 MATERIALS AND METHODS

### 2.1 Cell co‐culture

HUVEC and RA‐FLS were purchased from Saibaikang Biotechnology Co., Ltd. After digestion, the HUVEC were suspended in a culture medium, and 100 µL of the HUVEC suspension was added to each well. The edge wells were filled with sterile phosphate‐buffered saline (PBS) (Hyclone). Similarly, RA‐FLS were digested, and different volumes (100, 50, 25, and 10 µL) of the RA‐FLS suspension were added to a 96‐well plate and supplemented to a final volume of 100 µL with a complete culture medium. The treated groups received RA‐FLS supernatant with varying cell numbers, except for the control group. After incubation for a specific period, the culture medium was removed, and each well was supplemented with 100 µL of culture medium containing 10 µL of Cell Counting Kit‐8 (CCK‐8) solution. The plate was further incubated for 1 h. Finally, the optimal stimulation concentration of RA‐FLS was determined using the CCK‐8 assay.

### 2.2 Cell transfection

The siRNA and negative control siRNA (si‐NC), overexpression plasmid (pcDNA3.1‐LncRNA HOTAIR), and negative control (pcDNA3.1‐NC) of LncRNA HOTAIR were purchased from Gene Pharma. According to the different transfection genes, different genes were transfected into RA‐FLS by Lipofectamine 2000 (Thermo Fisher Scientific), and the cells were collected 48 h later.

### 2.3 Grouping and model preparation

After RA‐FLS grew to 60% confluency, they were divided into groups as follows: A: control group (HUVEC); B: model group (HUVEC + RA‐FLS), C: pcDNA3.1‐NC group (HUVEC + RA‐FLS+pcDNA3.1‐LncRNA HOTAIR‐NC), D: pcDNA3.1‐HOTAIR group (HUVEC + RA‐FLS+pcDNA3.1‐LncRNA HOTAIR), E: si‐NC group (HUVEC + RA‐FLS + si‐LncRNA HOTAIR‐NC), F: siRNA‐HOTAIR group (HUVEC + RA‐FLS + si‐LncRNA HOTAIR), G: siRNA‐HOTAIR‐Recilisib (PI3K/AKT activator) group (HUVEC + RA‐FLS + si‐LncRNA HOTAIR + Recilisib), H: siRNA‐NC‐Recilisib group (HUVEC + RA‐FLS + si‐LncRNA HOTAIR‐NC + Recilisib). Except for the control group A, 0.5 mL of suspension was added to Transwell chambers (Corning) for co‐culture with HUVEC for 48 h.

### 2.4 CCK‐8 assay

The RA‐FLS cell suspension was added to a 96‐well plate, with 100 μL per well. The cell culture plate was placed in an incubator and incubated overnight at 37°C with 5% CO_2_. After transfection, the cells were incubated for 48 h. The supernatant was collected. HUVEC were digested and resuspended, and 100 μL was added to each well, with the outer wells filled with sterile PBS. RA‐FLS were digested, resuspended, and added to each well, except for the control group, along with different treated RA‐FLS supernatants. After 48 h of incubation, the medium was removed, and each well was added with 100 μL of medium containing 10 μL CCK‐8, followed by an additional 1 h incubation. The absorbance values of each well at optical density 450 nm (Rayto; RT6100) were measured using an enzyme‐linked immunosorbent assay analyzer.

### 2.5 Quantitative real‐time polymerase chain reaction (qRT‐PCR)

Total RNA was extracted from HUVEC using a TRIzol reagent (Life Technologies). PrimeScript RT Reagent Kit (TaKaRa) was used for reverse transcription, and complementary DNA (cDNA) amplification was performed using 1 μg of total RNA as a template. The amplification was carried out by a protocol including steps of 95°C for 30 s, 95°C for 15 s (40 cycles), and 60°C for 30 s (40 cycles). The levels of gene expression were determined by fluorescence quantitative PCR (ABI; StepOnePlus) with β‐actin and U6 as internal controls, and quantified by the $2^{-∆∆C_{t}}$ method. The primers were provided by Sangon Biotech (Table 1).

**Table 1:** Primer sequence list.

Gene	Amplicon size (bp)	Forward primer (5′→3′)	Reverse primer (5′→3′)
Hu‐β‐actin	96	CCCTGGAGAAGAGCTACGAG	GGAAGGAAGGCTGGAAGAGT
Hu‐HOTAIR	193	GCAGTGGGGAACTCTGACTC	TTGAGAGCACCTCCGGGATA
Hu‐PIK3R2	99	CCAGCAGTACCAGGACAAGA	GCCTCAATTGCAGTACGCTT
Hu‐bFGF	168	AAGGAGTGTGTGCTAACCGT	CAGTTCGTTTCAGTGCCACA
hsa‐miR‐126‐3p		TCGTACCGTGAGTAATCGCG	AGTGCAGGGTCCGAGGTATT
hsa‐miR‐126‐3pRT		GTCGTATCCAGTGCAGGGTCCGAGGTATTCGCACTGGATACGACCGCATT	
Hu‐PI3K	138	TGTGGAGCTCGCTAAAGTCA	CACTCCTGCCCTAAATGGGA
Hu‐AKT	167	CTTTCGGCAAGGTGATCCTG	GTACTTCAGGGCTGTGAGGA
Hu‐VEGFA	97	TTTGGGAACACCGACAAACC	GGTGTCCTCATCCCTGTACC
Human‐U6	70	CTCGCTTCGGCAGCACA	AACGCTTCACGAATTTGCGT

### 2.6 Western blot (WB) analysis

Each well of a six‐well plate was added with 100 μL of radioimmunoprecipitation assay cell lysis buffer (Biosharp) and incubated for 30 min, followed by centrifugation at 12,000 rpm for 15 min. The supernatant containing total cellular proteins was collected and added to a 5X sodium dodecyl‐sulfate polyacrylamide gel electrophoresis protein denaturing loading buffer. Electrophoresis was performed for 90 min at 300 mA constant current, followed by transfer to a membrane. The membrane was blocked with Western blocking buffer at room temperature for 2 h, and then incubated with primary antibodies against VEGF (1:1000; Affinity), bFGF (1:500; Affinity), PI3K (1:500; Affinity), AKT (1:500; Affinity), and phosphorylated‐AKT (p‐AKT) (1:500; Affinity) overnight at 4°C. After washing with PBST three times, the membrane was incubated with horseradish peroxidase‐conjugated secondary antibodies at room temperature for 2 h. An electrochemiluminescence solution was applied to the membrane, and exposure conditions were adjusted according to the different intensities of luminescence. The film bands were analyzed using the “Image J” software.

### 2.7 Transwell assay

The cell concentration of each group was adjusted to 5 × 10^5^ cells/mL. Before use, the basal membrane of the Transwell chamber (LABSELECT) was hydrated. A hundred microliter of cell suspension was seeded in the upper chamber, while the lower chamber was filled with 600–800 µL of culture medium containing 10% serum. The chambers were then incubated in a 37°C cell culture incubator for 24 h. After removing the liquid from the upper chamber, the chambers were transferred to wells containing approximately 1 mL of 0.5% crystal violet staining solution (Beyotime). The chambers were stained at room temperature for 20 min.

### 2.8 Immunofluorescence (IF)

The cell plate was permeabilized for 15 min, followed by adding 3% bovine serum albumin (BSA) solution (Solarbio) for blocking at room temperature for 20 min. The BSA was removed from the samples, following which the AKT (1:200; CST), p‐AKT (1:200; Affinity), VEGF (1:200; Affinity), bFGF (1:200; Affinity), and PI3K (1:100; Abcam) antibodies were introduced and incubated at a temperature of 4°C in a humidified environment. All slices were then treated with a secondary antibody (1:500) and incubated in the dark at 37°C for 30 min. The cells were subjected to restaining with 4′,6‐diamidino‐2‐phenylindole for a duration of 5 min in the absence of light. Observation of the cells and collection of images was carried out using a fluorescence microscope (Evident).

### 2.9 Lumen formation experiment

Matrigel (Solarbio)was added to a 96‐well plate at a volume of 50 µL per well and incubated at 37°C for 1 h. Then, HUVEC and RA‐FLS were added to each well at a density of 1 × 105/mL in 200 µL of medium containing Matrigel. After incubation at 37°C for 8 h, the formed tubes were counted under a microscope (OLYMPUS) and analyzed using the Angiogenesis Analyzer plugin in the Image J software.

### 2.10 Dual‐Luciferase reporter assay

Twenty‐four hours before transfection, 293T cells were seeded in a 12‐well plate with a cell density of 2 × 10^5^ cells. Transfection was performed when the cell density reached 70%–80%. Serum‐free Dulbecco's modified Eagle's medium (DMEM) was used for transfection, and after 6 h, the medium was replaced with 10% fetal bovine serum DMEM without hygromycin. The cells were further cultured for 48 h. After that, 300 μL of cell lysis buffer was added, and the cells were centrifuged at 4°C and 12,000*g* for 10 min to collect the supernatant. The luciferase and Renilla luciferase reaction solutions were added to measure the activity of luciferase and Renilla luciferase, respectively.

### 2.11 Flow cytometry

Cells were collected and centrifuged at 1000 rpm for 5 min. After centrifugation, the cells were resuspended in 100 μL of PBS. Subsequently, Anti‐Human CD105, PE (Multi Sciences), and FITC Anti‐Human CD34 (Elabscience) were added to the cells in respective volumes of 5 μL each. The cells were then incubated for 20 min without exposure to light. Following the incubation, the cells were filtered and subjected to flow cytometry analysis using the Agilent NovoCyte flow cytometer. The light source utilized was an excitation light at 488 nm, and the filter settings were as follows: emission filter at 530/30 and 572/28. A minimum of 10,000 cells were collected at medium speed, and the light signals emitted by the cells were converted into electrical signals. The resulting data file was analyzed using the NovoExpress software (Agilent).

### 2.12 Statistical analysis

The experimental data were subjected to statistical analysis using SPSS 23.0 software (IBM Corp.) and image acquisition was carried out using GraphPad Prism 8.0 software (GraphPad Software). All data were presented as mean ± standard deviation. The normality of the data was assessed in SPSS using the Kolmogorov‐Smirnov test. If the data followed a normal distribution, Student's *t* test was employed for comparisons between two groups, and one‐way analysis of variance was used for comparisons among multiple groups. In cases where the data did not conform to a normal distribution, the interquartile range was utilized. *p* < .05 indicates statistical significance.

## 3 RESULTS

### 3.1 Optimal co‐culture ratio and duration, and the effect of lncRNA HOTAIR on HUVEC viability

**Figure 1:**
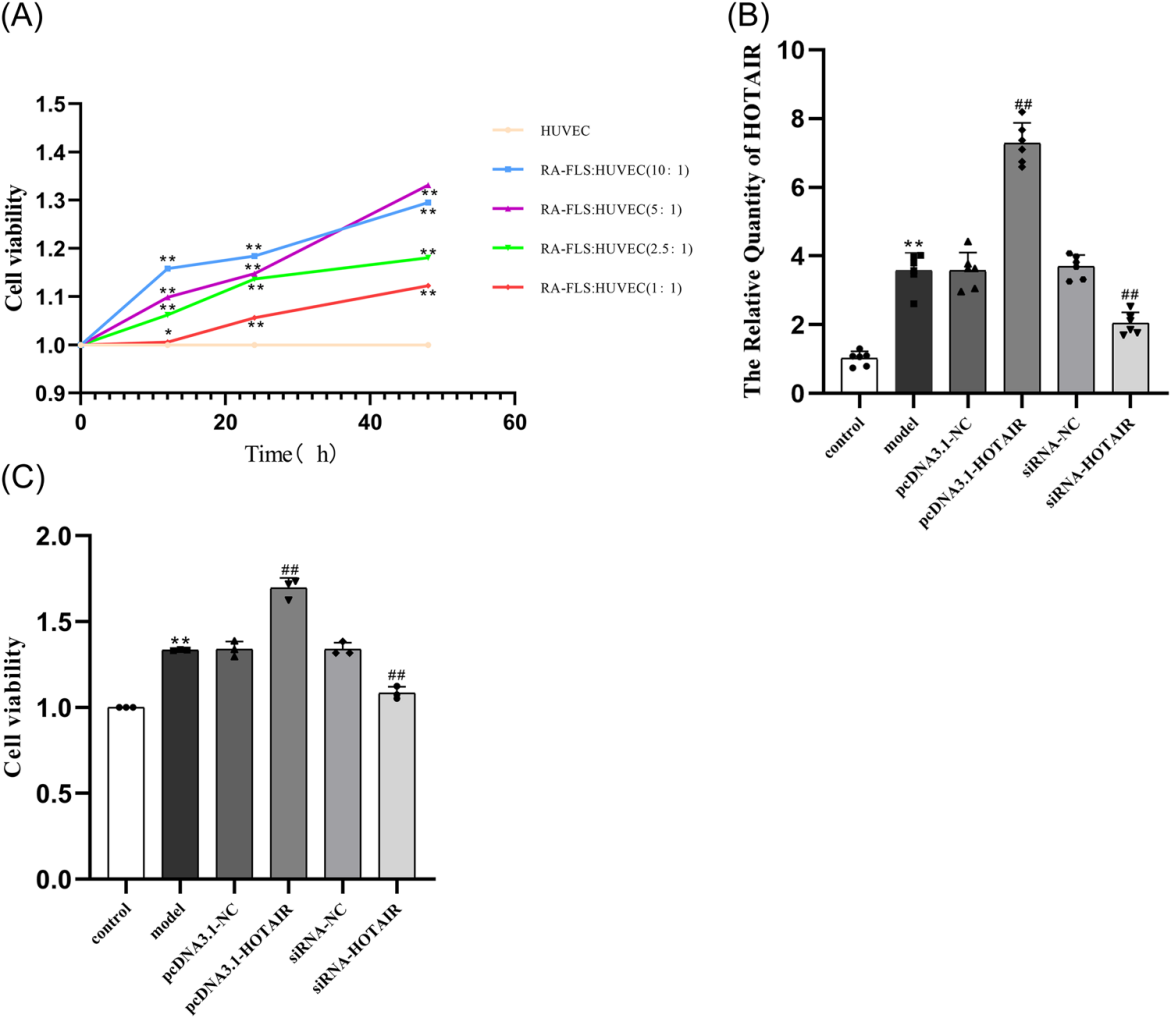
Screening of the optimal ratio and time of RA‐FLS and HUVEC and the effect of overexpression or knockdown of LncRNA HOTAIR on HUVEC viability. (A) Screening of the best ratio of RA‐FLS and HUVEC (5:1, 48 h), *p < .05, **p < .01. (B) Expression of HOTAIR in each group. (C) Proliferation capacity of HUVEC in each group. **p < .01 compared to the control group; ^##^ *p* < .01 compared to the model group. HUVEC, human umbilical vein endothelial cells; LncRNA, long‐chain noncoding RNA; NC, negative control; RA‐FLS, rheumatoid arthritis fibroblast‐like synoviocytes; siRNA, small interference RNA.

HUVEC were treated with various concentrations of RA‐FLS. The optimal stimulation concentration of RA‐FLS (5:1, 48 h) was determined by the CCK‐8 assay (Figure 1A). We successfully transfected overexpression plasmids or siRNA targeting LncRNA HOTAIR into RA‐FLS. Following transfection, HUVEC were stimulated with RA‐FLS. The expression of LncRNA HOTAIR in HUVEC was subsequently assessed using qRT‐PCR (Figure 1B). The expression of LncRNA HOTAIR in the model group of HUVEC was significantly higher than that in the control group. (*p* < .01, Figure 1B). Compared with the control group, the cell viability of the model group increased significantly (*p* < .01). Compared with the model group, the ability of cell proliferation in pcDNA3.1‐HOTAIR group was significantly increased, while that in siRNA‐HOTAIR group was significantly decreased (*p* < .01, Figure 1C).

### 3.2 LncRNA HOTAIR can regulate the migration, lumen formation ability, and levels of vascular endothelial markers in RA‐FLS‐stimulated HUVEC

**Figure 2:**
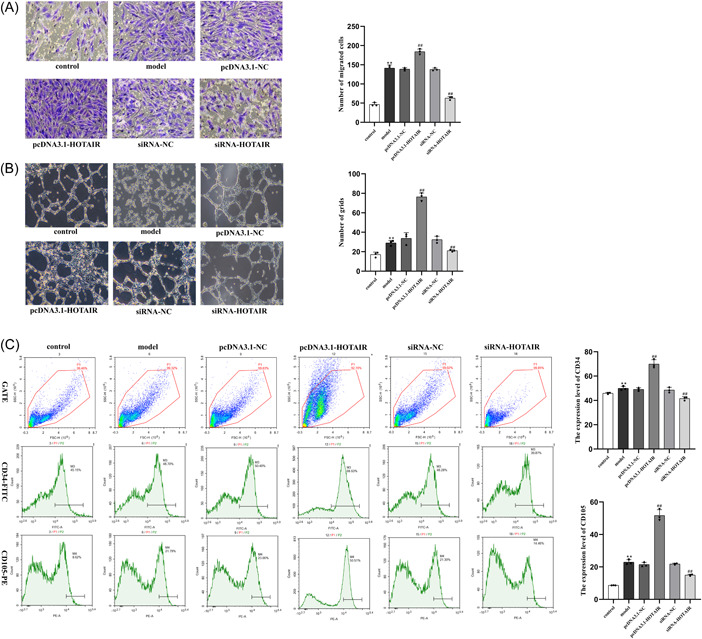
Regulation of the migration, tube formation ability, and vascular endothelial markers levels of RA‐FLS‐stimulated HUVEC by LncRNA HOTAIR. (A) Transwell assay to analyze the effect of LncRNA HOTAIR on HUVEC migration (scale bar, 100 μm, ×200). (B) Tube formation assay to analyze the effect of LncRNA HOTAIR on HUVEC tube formation ability (scale bar, 50 μm, ×100). (C) Flow cytometry to detect the expression levels of CD34 and CD105; **p < .01 compared to the control group; ^##^ *p* < .01 compared to the model group. HUVEC, human umbilical vein endothelial cells; LncRNA, long‐chain noncoding RNA; NC, negative control; RA‐FLS, rheumatoid arthritis fibroblast‐like synoviocytes; siRNA, small interference RNA.

We used a transwell assay to assess the migratory capability and conducted a tube formation assay to evaluate the tube‐forming ability of HUVEC. Compared with the control group, the cell migration and tube‐forming ability of the model group were significantly improved (*p* < .01). Compared with the model group, the results showed that the pcDNA3.1‐HOTAIR group exhibited higher cell migration and tube‐forming ability(*p* < .01). Conversely, the siRNA‐HOTAIR group showed lower cell migration and tube‐forming ability (*p* < .01, Figure 2A,B). We measured the expression levels of CD34 and CD105 using flow cytometry. Compared to the control group, the model group showed increased expression of CD34 and CD105 (*p* < .01). Compared to the model group, the pcDNA3.1‐HOTAIR group exhibited significantly increased expression of CD34 and CD105 (*p* < .01). Conversely, the siRNA‐HOTAIR group showed decreased levels of CD34 and CD105 (*p* < .01, Figure 2C).

### 3.3 LncRNA HOTAIR can regulate the expression levels of miR‐126‐3p and PIK3R2 in HUVEC stimulated by RA‐FLS

**Figure 3:**
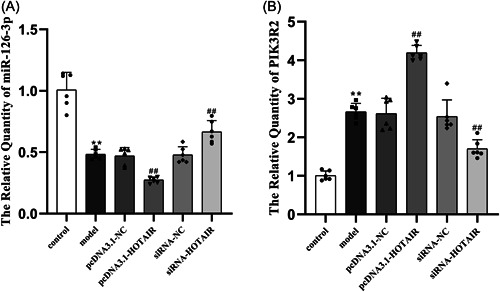
The effect of LncRNA HOTAIR on the expression levels of miR‐126‐3p and PIK3R2 genes. (A) qRT‐PCR was used to measure the expression level of miR‐126‐3p and (B) PIK3R2 gene in HUVEC stimulated by RA‐FLS. **p < .01 compared to the control group; ^##^ *p* < .01 compared to the model group. HUVEC, human umbilical vein endothelial cells; LncRNA, long‐chain noncoding RNA; NC, negative control; PIK3R2, phosphoinositide‐3‐kinase regulatory subunit‐2; qRT‐PCR, quantitative real‐time polymerase chain reaction; RA‐FLS, rheumatoid arthritis fibroblast‐like synoviocytes; siRNA, small interference RNA.

qRT‐PCR analysis revealed that, compared to the control group, the model group exhibited a significant decrease in miR‐126‐3p expression (*p* < .01) and a significant increase in PIK3R2 expression (*p* < .01). Compared to the model group, the pcDNA‐HOTAIR group showed a significant decrease in miR‐126‐3p expression (*p* < .01) and a significant increase in PIK3R2 expression (*p* < .01). In contrast, the siRNA‐HOTAIR group showed the opposite trend (*p* < .01, Figure 3A,B).

### 3.4 The targeting relationship among LncRNA HOTAIR, miR‐126‐3p, and PI3K2R

**Figure 4:**
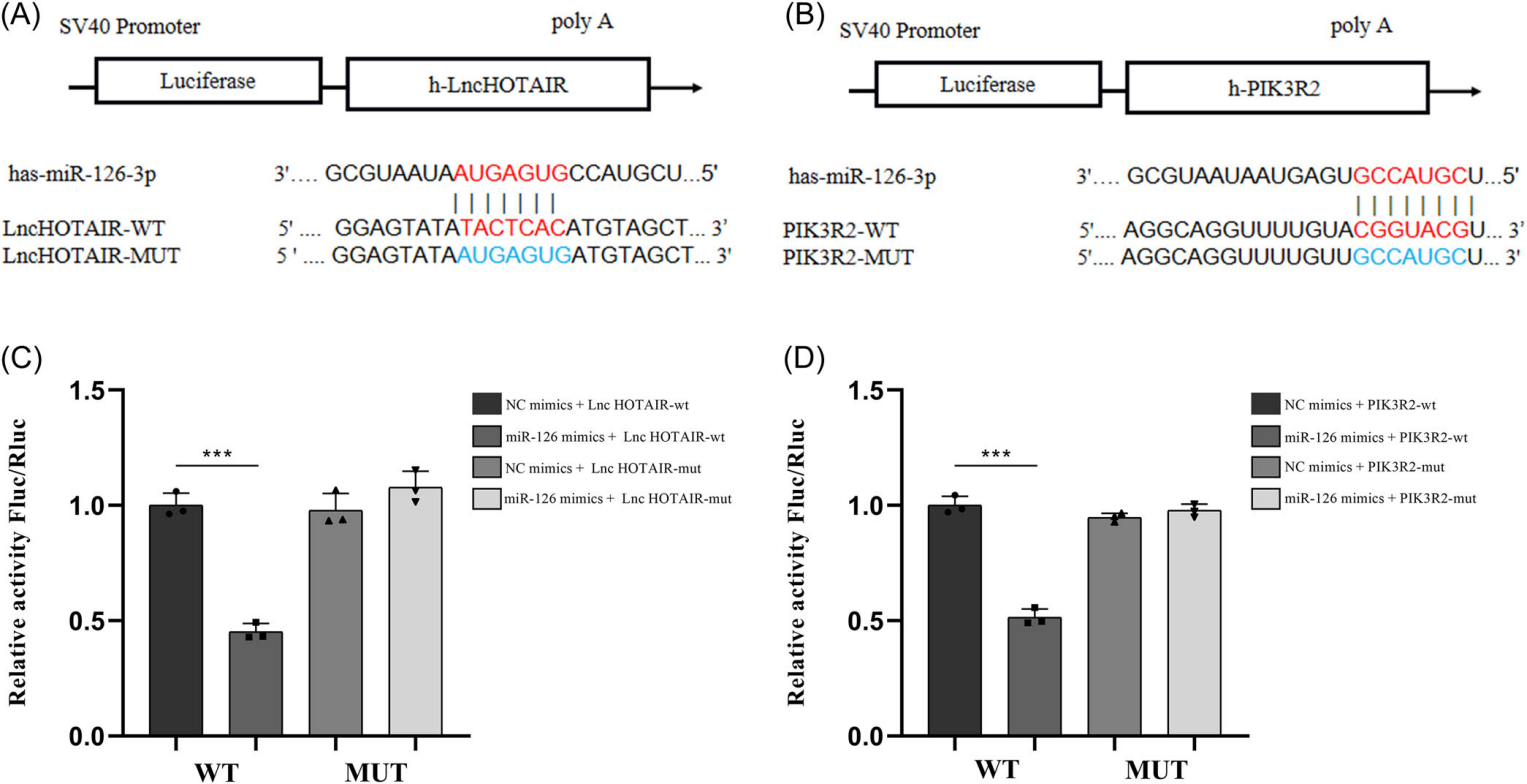
The prediction by bioinformatics software and the double‐luciferase reporter gene detection experiment. (A) Schematic diagram of the binding site of hsa‐miR‐126‐3p and h‐LncHOTAIR. (B) Schematic diagram of the binding site of hsa‐miR‐126‐3p and h‐PIK3R2. (C) Double‐luciferase reporter gene detection of the interaction between hsa‐miR‐126‐3p and h‐LncHOTAIR. (D) Double‐luciferase reporter gene detection of the interaction between hsa‐miR‐126‐3p and h‐PIK3R2. ***p < .001. LncRNA, long‐chain noncoding RNA; miRNA, microRNA; MUT, mutant; NC, negative control; PIK3R2, phosphoinositide‐3‐kinase regulatory subunit 2; WT, wild type.

Using bioinformatics software, the binding sequences between LncRNA HOTAIR and miR‐126‐3p, as well as miR‐126‐3p and PIK3R2 were predicted. Gene templates and mutant templates for the binding sequence of LncRNA HOTAIR with the 3′ untranslated region (3′UTR) and the binding sequence of PIK3R2 with the 3′UTR were obtained through gene synthesis. These templates were then inserted into a luciferase reporter gene vector to form dual‐luciferase reporter gene expression plasmids (Figure 4A,B). Results from the dual‐luciferase reporter gene detection experiment showed that after adding miR‐126 mimics, the fluorescence enzyme activity of LncRNA HOTAIR‐WT decreased (*p* < .001), while after adding miR‐126‐3p mimics, the fluorescence enzyme activity of PIK3R2‐WT also decreased (*p* < .001). These results suggested a targeting relationship between LncRNA HOTAIR and miR‐126‐3p, as well as between miR‐126‐3p and PIK3R2 (Figure 4C,D)

### 3.5 LncRNA HOTAIR/miR‐126‐3p/PIK3R2 axis regulates the expression of angiogenic factors and PI3K/AKT pathway

**Figure 5:**
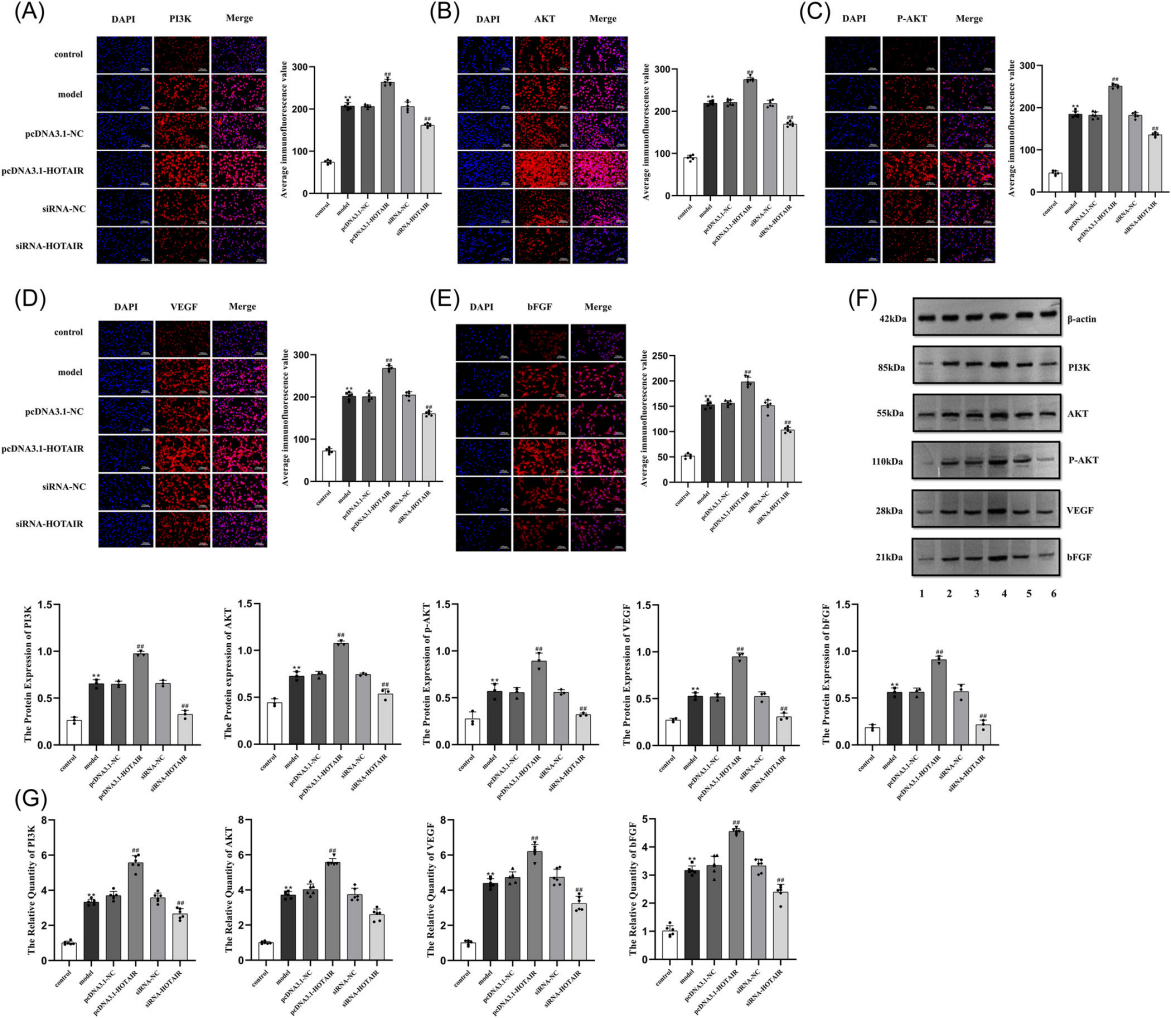
LncRNA HOTAIR/miR‐126/PIK3R2 axis regulates the expression of angiogenic factors and PI3K/AKT pathway genes. (A–F) The protein expressions of PI3K, AKT, p‐AKT, VEGF, and bFGF in each group were detected by immunofluorescence and Western blot (scale bar, 100 μm, ×200). (G) Detection of PI3K, AKT, and VEGF and bFGF gene expression by qRT‐PCR. **p < .01 compared to the control group; ^**##**^ *p* < .01 compared to the model group. (1. control; 2. model; 3. pcDNA3.1‐NC; 4. pcDNA3.1‐HOTAIR; 5. siRNA‐NC; 6. siRNA‐HOTAIR.) AKT, protein kinase B; bFGF, basic fibroblast growth factor; DAPI, 4′,6‐diamidino‐2‐phenylindole; HUVEC, human umbilical vein endothelial cells; LncRNA, long‐chain noncoding RNA; miRNA, microRNA; NC, negative control; P‐AKT, phosphorylated AKT; PI3K, phosphoinositide 3‐kinase; PIK3R2, phosphoinositide‐3‐kinase regulatory subunit‐2; qRT‐PCR, quantitative real‐time polymerase chain reaction; RA‐FLS, rheumatoid arthritis fibroblast‐like synoviocytes; siRNA, small interference RNA; VEGF, vascular endothelial growth factor.

To further verify the regulatory effect of LncRNA HOTAIR/miR‐126‐3p/PIK3R2 axis on angiogenic factor and PI3K/AKT pathway, IF and WB were used to detect the protein levels of PI3K, AKT, p‐AKT, VEGF, and bFGF, and qRT‐PCR was used to detect the levels of PI3K, AKT, VEGF, and bFGF genes. The results showed that the overexpression of LncRNA HOTAIR increased the levels of PI3K, AKT, p‐AKT, VEGF, and bFGF proteins in HUVEC (*p* < .01). On the contrary, siRNA‐HOTAIR has the opposite effect (*p* < .01, Figure 5A–F). qRT‐PCR results showed that overexpression of LncRNA HOTAIR promoted the expression of PI3K, AKT, VEGF, and bFGF genes in HUVEC (*p* < .01), while silencing had the opposite effect (*p* < .01, Figure 5G).

### 3.6 PI3K/AKT activator can reverse the effect of silenced LncRNA HOTAIR on HUVEC

**Figure 6:**
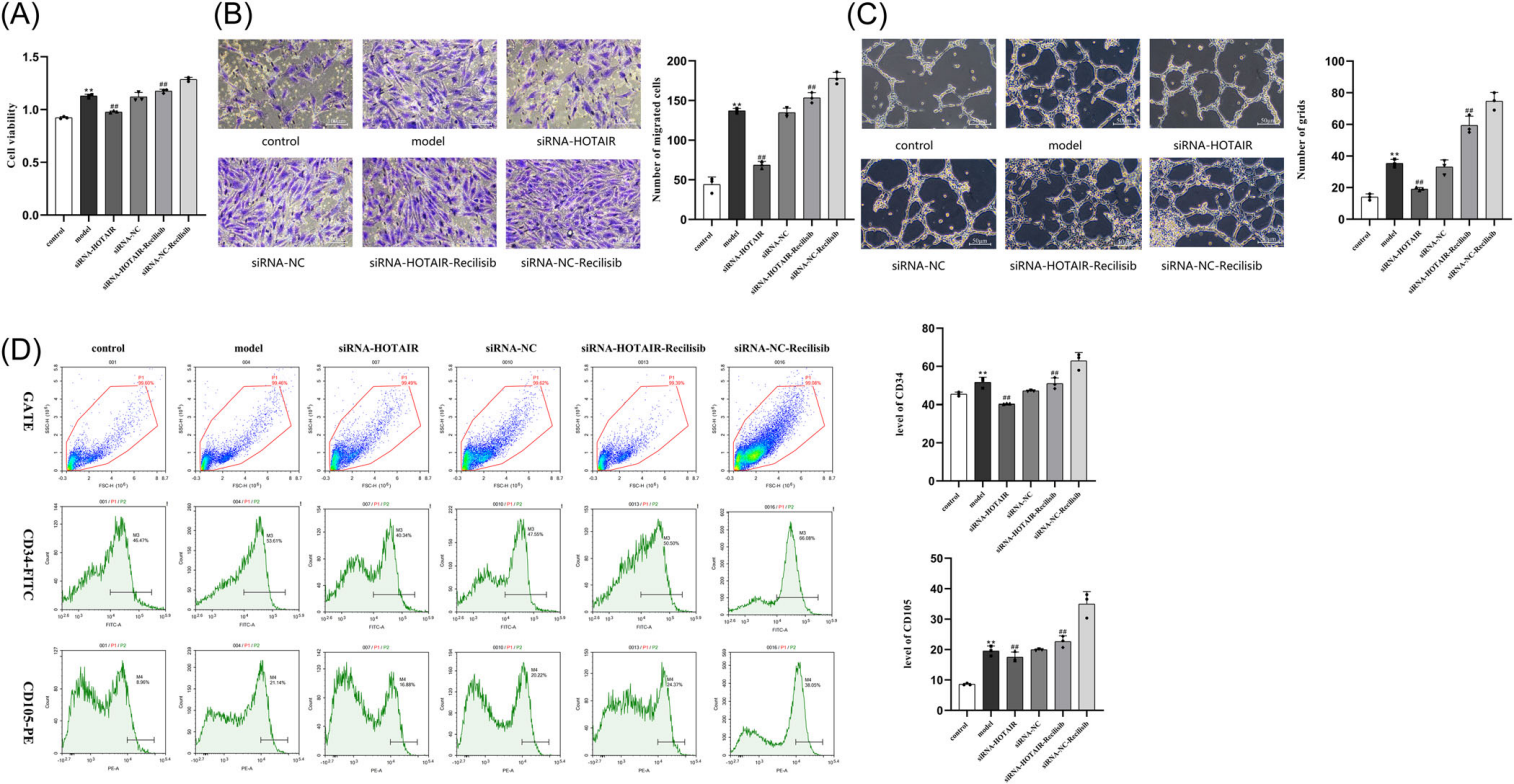
PI3K/AKT activator can reverse the effects of silenced LncRNA HOTAIR on the proliferation, migration, tube formation, and CD34/CD105 expression of HUVEC. (A) Cell proliferation ability of HUVEC. (B) Cell migration ability of HUVEC (scale bar, 100 μm, ×200). (C) Tube formation ability of HUVEC (scale bar, 50 μm, ×100). (D) Expression levels of CD34 and CD105 of HUVEC. **p < .01 compared to the control group; ^##^ *p* < .01 compared to the model group. CD34, cluster of differentiation 34; CD105, cluster of differentiation 105; HUVEC, human umbilical vein endothelial cells; LncRNA, long noncoding RNA; NC, negative control; PI3K/AKT, phosphoinositide 3‐kinase/protein kinase B; RA‐FLS, rheumatoid arthritis fibroblast‐like synoviocytes; siRNA, small interference RNA.

Finally, we want to know whether the PI3K activator can reverse the effect of silent LncRNA HOTAIR on HUVEC. Compared with the model group, the proliferation, migration, and tube formation ability of cells in the siRNA‐HOTAIR group were significantly inhibited (*p* < .01), while the siRNA‐HOTAIR‐Recilisib group was significantly increased (*p* < .01, Figure 6A–C). Flow cytometry analysis showed that the expression of CD34 and CD105 was decreased in the siRNA‐HOTAIR group (*p* < .01), while the expression of CD34 and CD105 was significantly increased in the siRNA‐HOTAIR‐Recilisib group (*p* < .01, Figure 6D). In conclusion, we concluded that PI3K activator could reverse the effects of silent LncRNA HOTAIR on proliferation, migration, tube formation, and CD34 and CD105 expression of HUVEC.

## 4 DISCUSSION

As research into RA continues, it has been observed that RA could be classified as a “vasculogenic disease” because of its association with active tissue neovascularization.^21^ Synovitis and vasculitis are important pathological changes of RA, which can trigger angiogenesis under the conditions of inflammation, immune imbalance, and hypoxia.^22^ VEGF and bFGF can promote angiogenesis and vascular remodeling, increasing the blood supply to the synovium and providing more nutrients and cytokines to inflammatory cells, exacerbating the development of arthritis.^23^, ^24^ FLS and HUVEC can produce various cytokines and chemokines, regulating synovial inflammation and angiogenesis.^25^ Abnormal activation and proliferation of FLS are observed in RA patients, and by secreting inflammatory and proangiogenic factors, they promote endothelial cell activation to induce inflammation, thereby forming a vicious cycle between angiogenesis and synovial inflammation in RA patients.^26^ Therefore, increasing attention is being focused on antiangiogenic therapies for RA.

Most lncRNAs do not encode proteins but participate in cellular processes such as proliferation and differentiation, acting as transcriptional regulators and competitively binding microRNAs (miRNAs) to regulate the levels of target proteins.^27^ LncRNA HOTAIR, derived from the HOX gene locus, can directly activate the transcription of vascular endothelial growth factor A, promoting angiogenesis.^28^ Compared to the model group, we found that in the pcDNA3.1‐HOTAIR group, HUVEC showed significantly increased proliferation, invasion, and tube formation ability, as well as elevated expression levels of CD34 and CD105. Conversely, in the siRNA‐HOTAIR group, HUVEC exhibited significantly decreased proliferation, invasion, tube formation ability, and expression levels of CD34 and CD105. These findings suggest the potential role of LncRNA HOTAIR in angiogenesis in RA.

The expression of angiogenic factor by LncRNA HOTAIR is related to the activation of PI3K/AKT pathway. In the report of RA synovial angiogenesis, it is found that the activation of PI3K/AKT signal pathway can promote the proliferation, migration, and angiogenesis of vascular endothelial cells.^29^ It was found that the expression of PI3K, AKT, VEGF, and bFGF genes in HUVEC in the pcDNA3.1‐HOTAIR group was significantly higher than that in the model group, while the expression of PI3K, AKT, VEGF, and bFGF genes in HUVEC in siRNA‐HOTAIR group was significantly decreased. IF and WB analysis showed that the expression of PI3K, AKT, p‐AKT, VEGF, and bFGF in HUVEC in pcDNA3.1‐HOTAIR group was significantly higher than that in model group, while the expression of PI3K, AKT, p‐AKT, VEGF, and bFGF in HUVEC in siRNA‐HOTAIR group was significantly lower than that in model group. The results of the salvage experiment further showed that the addition of PI3K/AKT activator could reverse the inhibitory effect of silent LncRNA HOTAIR on HUVEC.

miR‐126 is a posttranscriptional regulator of gene expression and an important member of the miRNA family, which can regulate the PI3K/AKT signaling pathway by affecting the PIK3R2 gene, thereby playing a role in angiogenesis, cell proliferation, differentiation, and migration.^19^ It has been reported that LncRNA HOTAIR regulates renal cell carcinoma angiogenesis through the miR‐126/EGFL7 axis.^19^ LncRNA HOTAIR overexpression inhibits the Wnt/β‐catenin signaling pathway by regulating the miR‐126/Klotho/SIRT1 axis, thereby weakening phosphate‐induced vascular calcification.^30^ The LncRNA HOTAIR/miR‐126/SCEL axis mediates angiogenesis and contributes to wound healing in burn injuries.^31^ In the qRT‐PCR experiments, we observed a marked upregulation of miR‐126‐3p in HUVEC, which was accompanied by a notable downregulation of LncRNA HOTAIR and PIK3R2 expression. Conversely, when miR‐126‐3p levels were significantly decreased, there was a pronounced upregulation of LncRNA HOTAIR and PIK3R2 expression. Further bioinformatics analysis and luciferase assay confirmed the targeting relationship between LncRNA HOTAIR and miR‐126‐3p, and also between miR‐126‐3p and PIK3R2. The data suggest that LncRNA HOTAIR participates in angiogenesis in the RA synovial membrane by regulating the PI3K/AKT pathway through the miR‐126‐3p/PIK3R2 axis. The data suggest that LncRNA HOTAIR participates in angiogenesis in the RA synovial membrane by regulating the PI3K/AKT pathway through the miR‐126‐3p/PIK3R2 axis.

Neovascularization has been extensively studied in cancer tumor research, but less is known about it in RA. Therefore, we conducted an in vitro experiment to elucidate the potential molecular mechanisms for treating RA by focusing on neovascularization. However, the experiment has some limitations and incompleteness. First, more research is needed to elucidate the genes involved in the LncRNA HOTAIR axis, and the effect of miR‐126 and PIK3R2 overexpression or knockdown needs to be further validated from the perspective of competitive endogenous RNA mechanism for a more comprehensive analysis. Second, further animal experiments and clinical trials are required to verify whether this signaling axis plays a role in RA.

In summary, our study sheds light on the potential role of neovascularization in the pathogenesis of RA. The LncRNA HOTAIR/miR‐126‐3p/PIK3R2 signaling axis may contribute to RA neovascularization and provide novel insights and potential targets for therapeutic intervention in RA.

## AUTHOR CONTRIBUTIONS

Feifei Liu and Yuan Wang contributed to the study design. Feifei Liu contributed to data analysis, wrote the first draft, and revised the manuscript. Yanqiu Sun and Dan Huang supervised the project and contributed to the manuscript revision. All authors reviewed and accepted the content of the final manuscript.

## CONFLICT OF INTEREST STATEMENT

The authors declare no conflict of interest.

## Supporting information

Supporting information.

## Data Availability

*Data availability*: The data availability complies with the requirements of the journal. *Materials and Methods*: All materials and methods used in the experiments are detailed in the paper. *Data source*: The original data for this study can be obtained from (name or link of the data repository). *Data processing*: The data processing methods for this study are detailed in the paper.
