# Supplementary material for: LncRNA HOTAIR regulates the PI3K/AKT pathway via the miR‐126‐3p/PIK3R2 axis to participate in synovial angiogenesis in rheumatoid arthritis
Source: Immun Inflamm Dis. 2023 Oct 27;11(10):e1064. doi: 10.1002/iid3.1064 (PMC10604569; doi:10.1002/iid3.1064)
Supplement: Supplementary file 1 — Supporting information. [file IID3-11-e1064-s001.docx]

**Experimental Materials And Methods**

1. **Cell culture experiment....................................................23**

**2. Cell transfection experiment............................................39**

**3.Co-culture experiment.......................................................56**

**4.Cell proliferation experiment............................................77**

**5.Transwell Migration Assay................................................96**

**6.Lumen formation experiment...........................................119**

**7.Real-time fluorescence quantitative PCR experiment....133**

**8.Experimental report on double luciferase........................170**

**9.Experimental report of immunofluorescence...................216**

**10.Western blot experiment report.......................................245**

**11.Flow cytometry detection experiment..............................323**

**12.Rescue experiment.............................................................337**

**1.Cell Culture Experiment**

**1.1 Experimental subjects**

HUVEC；RA-FLS

**1.2 Experimental step**

RA-FLS were seeded in a six-well plate and incubated overnight. Upon reaching 60% confluency, they were divided into six groups: B: normal RA-FLS, C: over-expressing negative control (NC), D:over-expressing pcDNA3.1-LncRNA HOTAIR plasmid, E: interfering NC, F: transfecting small interfering RNA targeting LncRNA HOTAIR.G: Transfect with small interfering RNA targeting LncRNA HOTAIR and 50μm PI3K activator Recilisib.H: Transfect with interfering NC and 50μm PI3K activator Recilisib.After 48 hours of transfection, the cells from the six groups were digested, centrifuged, and resuspended to a density of 5*10^6^/ml.HUVEC were seeded at a density of 5*10^5^ in a six-well plate and incubate overnight. Except for group A, 0.5ml of the suspension was added to the Transwell chamber and placed on top of the HUVEC for co-culture for 48 hours under regular culture conditions.

**1.3The main reagents of the experiment**

| **Reagent** | **Manufacturer** | **Item number** |
| --- | --- | --- |
| DMEM high sugar medium  Lipofectamine™ 2000 | Serveicebo  Thermo Fisher | G4512  11668-027 |

**1.4 Experimental equipment**

| **Name** | **Manufacturer** | **Model** |
| --- | --- | --- |
| Liquid transfer gun | Thermo scientific | FINNPIPETTE®F3 |
| Inverted microscope | OLYMPUS | CKX53 |
| Centrifuge machine | Anhui Jiawen instrument | JW-1024 |
| Incubator | Thermo scientific | 3111 |
| Super clean workbench | Shanghai Lichen instrument | SW-CJ-2D |
| Digital display water  bath | Shanghai Lichen Bangxi instrument | HH-4 |

**2. Cell transfection experiment**

**2.1 Experimental Step**

The cells were passaged into five wells of a 6-well plate at a ratio of 1 full T25 flask after normal digestion and placed in a cell culture incubator for 24 hours. Transfection was performed the following morning when the well's cell density reached 70%. For siRNA transfection, 1000 rpm for 1 minute centrifugation of the tube containing dry RNA powder and addition of 125μl of DEPC water to the siRNA were conducted. Transfection reagent preparation was as follows: A: Dissolving 5μl of siRNA and NC separately in 250μl of serum-free medium, B: Dissolving 10 μl of Lipofectamine 2000 in 1000μl of serum-free medium, mixing, and letting it stand at room temperature for 5 minutes. C: Splitting B into 2 parts, adding them to A, mixing, and leaving at room temperature for 20 minutes. The 6-well plate was rinsed 2-3 times with serum-free medium. Adding the mixed culture medium (500μl) to the 6-well plate, gently shaking for 1-2 minutes, adjusting the volume to 2 ml, and placing it in a culture incubator for 4 hours. Afterward, replacing it with the original complete medium and continuing the culture. After 48 hours, cells were collected for qPCR analysis to assess transfection efficiency.

**2.2 The main reagents of the experiment**

| **Reagent** | **Manufacturer** | **Item number** |
| --- | --- | --- |
| CCK8 detection kit | Servicebio | G4103 |
| trypsin | Beyotime | C0201 |
| HUVEC special medium | iCell | iCell-h110-001b |
| DMEM high sugar medium | Serveicebo | G4512 |
| PBS | Hyclone | SH30256.01 |
| FBS | BI | 04-001-1ACS |

**2.3 Experimental equipment**

| **Name** | **Manufacturer** | **Model** |
| --- | --- | --- |
| Liquid transfer gun | Thermo scientific | FINNPIPETTE®F3 |
| Inverted microscope | OLYMPUS | CKX53 |
| Centrifuge machine | Anhui Jiawen instrument | JW-1024 |
| Incubator | Thermo scientific | 3111 |
| Super clean workbench | Shanghai Lichen instrument | SW-CJ-2D |
| Digital display water  bath | Shanghai Lichen Bangxi instrument | HH-4 |

**3.Co-culture experiment**

**3.1 Experimental Step**

After normal digestion of HUVEC, they are resuspended in culture medium at a cell density of 1*10^5^. HUVEC suspension is prepared and gently mixed.100μl is added to each well, and the edge wells are filled with sterile PBS. After normal digestion of RA-FLS, they are resuspended in culture medium at a cell density of 1*10^6^. The RA-FLS suspension is prepared and gently mixed. Then, 100μl, 50μl, 25μl, and 10μl of cell suspension are added to the wells of a 96-well plate, and they are topped up with complete culture medium to a final volume of 100μl. The seeded cell culture plates are placed in an incubator and are incubated overnight at 37°C with 5% CO_2_. In the treatment groups, except for the normal group, the supernatant containing different numbers of RA-FLS is added. After different time periods of culturing, the culture media are removed, and 100μl of culture medium containing 10μl of CCK8 is added to each well. Then, they are incubated for 1 hour. The absorbance at OD450nm is measured using an enzyme-linked immunosorbent assay (ELISA) reader for each well. Blank wells are set up with culture medium and CCK8.

**3.2 The main reagents of the experiment**

| **Reagent** | **Manufacturer** | **Item number** |
| --- | --- | --- |
| CCK8 detection kit | Servicebio | G4103 |
| trypsin | Beyotime | C0201 |
| HUVEC special medium | iCell | iCell-h110-001b |
| DMEM high sugar medium | Serveicebo | G4512 |
| PBS | Hyclone | SH30256.01 |
| FBS | BI | 04-001-1ACS |

**3.3 Experimental equipment**

| **Name** | **Manufacturer** | **Model** |
| --- | --- | --- |
| Liquid transfer gun | Thermo scientific | FINNPIPETTE®F3 |
| Inverted microscope | OLYMPUS | CKX53 |
| Centrifuge machine | Anhui Jiawen instrument | JW-1024 |
| Incubator | Thermo scientific | 3111 |
| Super clean workbench | Shanghai Lichen instrument | SW-CJ-2D |
| Digital display water  bath | Shanghai Lichen Bangxi instrument | HH-4 |
| Enzyme labeling  instrument | Rayto | RT6100 |

**3.4 Result interpretation**

According to the cell viability of each group, it is suggested that the ratio of synovial fibroblasts to HUVEC should be selected for 48 hours.

**4.Cell proliferation experiment**

**4.1 Experimental Step**

After preparing the RA-FLS suspension, gently mix it and add the 100ul cell suspension to the 96-well plate.The inoculated cell culture plate was placed into the incubator overnight and incubated at 37 ℃ with 5% CO_2_. RA-FLS were transfected for 48 hours. The supernatant of cells in each group was collected. After normal digestion, HUVEC were re-suspended in culture medium, and the cell density was 1*10^5^. After the HUVEC suspension was prepared, gently mix it, and 100ul was added to each hole, and the marginal hole was filled with aseptic PBS. After normal digestion, RA-FLS were re-suspended with culture medium, and the cell density was 0.5*10^6^. The supernatants of different treatments of RA-FLS were added to other groups except the normal group. After 48 hours of cultivation, remove the culture media and add 100μl of culture medium containing 10μl of CCK8 to each well, then continue incubating for 1 hour. The absorbance of each hole was measured at OD450nm. At the same time, blank holes (culture medium, CCK8) were set up.

**4.2 The main reagents of the experiment**

| **Reagent** | **Manufacturer** | **Item number** |
| --- | --- | --- |
| CCK8 detection kit | Servicebio | G4103 |
| trypsin | Beyotime | C0201 |
| HUVEC special medium | iCell | iCell-h110-001b |
| DMEM high sugar medium | Serveicebo | G4512 |
| PBS | Hyclone | SH30256.01 |
| FBS | BI | 04-001-1ACS |

**4.3 Experimental equipment**

| **Name** | **Manufacturer** | **Model** |
| --- | --- | --- |
| Liquid transfer gun | Thermo scientific | FINNPIPETTE®F3 |
| Inverted microscope | OLYMPUS | CKX53 |
| Centrifuge machine | Anhui Jiawen instrument | JW-1024 |
| Incubator | Thermo scientific | 3111 |
| Super clean workbench | Shanghai Lichen instrument | SW-CJ-2D |
| Digital display water  bath | Shanghai Lichen Bangxi instrument | HH-4 |
| Enzyme labeling  instrument | Rayto | RT6100 |

**4.4 Result interpretation**

Mapping according to the cell viability of each group

**5.Transwell Migration Assay**

**5.1 Experimental Step**

Before use, the Transwell chambers were filled with an appropriate amount of serum-free culture medium and incubated at 37°C to hydrate the basement membrane. The cells in each group were digested, washed once with PBS and serum-free culture medium, suspended in serum-free culture medium, counted, and adjusted to a concentration of 5*10^5^/ml. In the lower chamber (i.e., the bottom of the 24-well plate), 600-800μl of culture medium containing 10% serum was added, and in the upper chamber, 100μl of cell suspension was added. The cells were then incubated in the cell culture incubator for 24 hours.With caution, the Transwell chambers were removed, and the liquid in the upper chamber was aspirated and transferred to wells containing approximately 1 ml of 4% paraformaldehyde, and the cells were fixed at room temperature for 20 minutes. The Transwell chambers were then removed again, and the fixative liquid in the upper chamber was aspirated and transferred to wells containing approximately 1 ml of 0.5% crystal violet, and the cells were stained at room temperature for 20 minutes. The chambers were gently rinsed several times with distilled water, and then the chambers were removed, and the liquid in the upper chamber was aspirated. A moist cotton swab was carefully used to wipe off the cells from the surface of the upper chamber membrane.The chambers were air-dried, and cell counts were conducted in three random fields under a microscope, and the results were recorded.

**5.2 The main reagents of the experiment**

| **Reagent** | **Manufacturer** | **Item number** |
| --- | --- | --- |
| Transwell  Small chamber | LABSELECT | 14342 |
| Crystal violet | Beyotime | C0121 |
| Fixed liquid | Serveicebo | G1101 |

**5.3 Experimental equipment**

| **Name** | **Manufacturer** | **Model** |
| --- | --- | --- |
| Liquid transfer gun | Thermo scientific | FINNPIPETTE®F3 |
| Inverted microscope | OLYMPUS | CKX53 |
| Centrifuge machine | Anhui Jiawen instrument | JW-1024 |
| Incubator | Thermo scientific | 3111 |
| Super clean workbench | Shanghai Lichen instrument | SW-CJ-2D |
| Digital display water  bath | Shanghai Lichen Bangxi instrument | HH-4 |

**5.4 Result interpretation**

Three visual fields were randomly selected to take pictures and cell count.

**6.Lumen formation experiment**

**6.1 Experimental Step**

Matrigel is thawed overnight at 4°C. Matrigel is pipetted vertically into pre-chilled 96-well cell culture plates at 4°C, with 50μl per well, using a cold pipette tip. The plates are then incubated at 37°C for 1 hour to allow the Matrigel to solidify. Cells from each group are digested, washed once with PBS and serum-free culture medium, suspended in complete culture medium, counted, and adjusted to a concentration of 1*10^5^/ml. The cells from each group are added to Matrigel-coated wells, with 200μl per well, and incubated in the cell culture incubator for 8 hours. Carefully remove the culture medium, wash once with PBS, add 200μl of PBS, randomly select three fields under a microscope for cell counting, and record the results.

**6.2 The main reagents of the experiment**

| **Reagent** | **Manufacturer** | **Item number** |
| --- | --- | --- |
| CCK8 detection kit | Servicebio | G4103 |
| trypsin | Beyotime | C0201 |
| HUVEC special medium | iCell | iCell-h110-001b |
| DMEM high sugar medium | Serveicebo | G4512 |
| PBS | Hyclone | SH30256.01 |
| FBS | BI | 04-001-1ACS |
| Matrigel Glue | Solarbio | M8371 |

**6.3 Experimental equipment**

| **Name** | **Manufacturer** | **Model** |
| --- | --- | --- |
| Liquid transfer gun | Thermo scientific | FINNPIPETTE®F3 |
| Inverted microscope | OLYMPUS | CKX53 |
| Centrifuge machine | Anhui Jiawen instrument | JW-1024 |
| Incubator | Thermo scientific | 3111 |
| Super clean workbench | Shanghai Lichen instrument | SW-CJ-2D |
| Digital display water  bath | Shanghai Lichen Bangxi instrument | HH-4 |

**6.4 Result interpretation**

Use the Angiogenesis Analyzer plug-in of Image J software for analysis.

**7.Real-time fluorescence quantitative PCR experiment**

**7.1 Experimental primer**

1）cDNA was taken as the template of fluorescence quantification, and the reaction system was as follows:

| **System** | **Volume** |
| --- | --- |
| 2×SYBR Green qPCR Master Mix  （High ROX） | 10uL |
| Forward Primer（10uM） | 0.4uL |
| Reverse Primer （10uM） | 0.4uL |
| cDNA | 3uL |
| RNase Free water | 6.2uL |
| Total | 20uL |

1. The reaction conditions are as follows:

| **Temperature** | **Time** | |
| --- | --- | --- |
| 95℃ | 30s | |
| 95℃ | 40 cycles | 15s |
| 60℃ |  | 30s |

3）Primers for each detection index:

| Gene | Amplicon Size  （bp） | Forward primer  （5'→3'） | Reverse primer  （5'→3'） | |
| --- | --- | --- | --- | --- |
| Hu-β-actin | 96 | CCCTGGAGAAGAGCTACGAG | GGAAGGAAGGCTGGAAGAGT | |
| Hu-HOTAIR | 193 | GCAGTGGGGAACTCTGACTC | TTGAGAGCACCTCCGGGATA | |
| Hu-PIK3R2 | 99 | CCAGCAGTACCAGGACAAGA | GCCTCAATTGCAGTACGCTT | |
| Hu-bFGF | 168 | AAGGAGTGTGTGCTAACCGT | CAGTTCGTTTCAGTGCCACA | |
| hsa-miR-126-3p |  | TCGTACCGTGAGTAATCGCG | AGTGCAGGGTCCGAGGTATT | |
| hsa-miR-126-3p RT |  | GTCGTATCCAGTGCAGGGTCCGAGGTATTCGCACTGGATACGACCGCATT | | |
| Hu-PI3K | 138 | TGTGGAGCTCGCTAAAGTCA | | CACTCCTGCCCTAAATGGGA |
| Hu-AKT | 167 | CTTTCGGCAAGGTGATCCTG | | GTACTTCAGGGCTGTGAGGA |
| Hu-VEGFA | 97 | TTTGGGAACACCGACAAACC | | GGTGTCCTCATCCCTGTACC |
| Human-U6 | 70 | CTCGCTTCGGCAGCACA | | AACGCTTCACGAATTTGCGT |

**7.2 Experimental Step**

The cell pellet was collected and subjected to 1 ml of TRIzol for lysis. Then, 0.2 mL of chloroform was added, vigorously shaken for 15 seconds, and left at room temperature for 5 minutes. Afterward, it was centrifuged at 12,000 rpm for 10 minutes at 4°C, and the supernatant (approximately 500μL) was transferred to another EP tube. Next, 0.5mL of pre-chilled isopropanol was added, gently mixed, and incubated on ice for 30 minutes.It was then centrifuged at 12,000 rpm for 15 minutes at 4°C, and the supernatant was discarded. Subsequently, 1 mL of pre-chilled 75% ethanol was added, followed by another centrifugation at 12000 rpm for 5 minutes at 4°C. The supernatant was again discarded, and this step was repeated.The RNA precipitate was air-dried at room temperature and then dissolved in 20-50μL of DEPC water. It was stored at -80°C for future use.To remove genomic DNA, a reaction was performed as follows: In a 0.2mL EP tube, total RNA (1µg) was mixed with 2.0μL of 5×gDNA Eraser Buffer, 1.0μL of gDNA Eraser, and DEPC water to make up a total volume of 10μL. The mixture was gently mixed and briefly centrifuged. It was then heated at 42°C for 2 minutes using a PCR machine, immediately followed by a 1-minute ice bath.In the same EP tube, PrimeScript RT Enzyme Mix I (1.0μL), RT Primer Mix (1.0μL), RNase Free dH2O(4.0μL), and RevertAid^TM^ M-MuLV Reverse Transcriptase (4.0μL) were added. The reaction mixture was incubated at 37°C for 15 minutes and then denatured at 85°C for 5 seconds.The resulting reaction mixture contained the cDNA and was stored at -20°C for future use.

**7.3 The main reagents of the experiment**

| **Reagent** | **Manufacturer** | **Item number** |
| --- | --- | --- |
| Trizol | Life technogies | 15596018 |
| Chloroform | Shanghai Suyi Chemical Reagent |  |
| Absolute ethanol | Shanghai Suyi Chemical Reagent |  |
| Isopropyl alcohol | Shanghai Suyi Chemical Reagent |  |
| DEPC-H_2_O | Generay Biotech | D1007 |
| 2×SYBR Green qPCR  Master Mix（High ROX） | Servicebio | G3322-05 |
| PrimeScript™RT reagent Kit  with gDNA Eraser | TaKaRa | RR047A |
| Primer synthesis | Sangon Biotech |  |

**7.3 Experimental equipment**

| **Name** | **Manufacturer** | **Model** |
| --- | --- | --- |
| ordinary PCR instrument | Bio-Rad | PTC-200 |
| low speed mini centrifuge | Haimen Kylin-Bell Lab Instruments | LX 300 |
| desktop high speed freezing centrifuge | Zhengzhou Honghua instrument | S-1-150S |
| micro pipette | Germany Eppendorf |  |
| fluorescence quantitative PCR instrument | ABI | StepOne Plus |
| microplate mini centrifuge | Hangzhou Aosheng instrument | MINI-P25 |
| ultra micro spectrophotometer | Nanjing Wuyi Technology | OD1000+ |

**7.4 Result interpretation**

1. The analysis method used in this experiment is Relative Quantification Study, and the calculation method is2-△△Ct.

2. The results include amplification curve, melting curve and relative expression, which are detailed in the analysis table of the results.

**8.Experimental report on double luciferase**

**8.1 Experimental Purpose**

1.Verify the targeting relationship between miR-126 and LncRNA HOTAIR .

2.Verify the targeting relationship between miR-126 and PIK3R2

**8.2 Experimental Principle**

Firefly Luciferase is a single subunit protein of 61KDa, which can catalyze the oxidation of luciferin to form a single subunit protein of oxyluciferin; Luciferase from sea kidney is a single subunit protein of 36KDa, which can catalyze the oxidation of coelenterin to coelenteramide.. Both of them can play a role without modification after translation. The strongest luminescence wavelength of Luciferin catalyzed by firefly luciferase is 560nm. The strongest luminescence wavelength of coelenterazine catalyzed by sea kidney luciferase is 465nm.

**8.3 Experimental grouping**

**miR-126 and LncRNA HOTAIR：**

NC-mimics + LncRNA HOTAIR-wt

miR-126- mimics + LncRNA HOTAIR-wt

NC mimics + LncRNA HOTAIR-mut

miR-126-mimics + LncRNA HOTAIR-mut

**miR-126 and PIK3R2：**

NC-mimics +PIK3R2 -wt

miR-126- mimics + PIK3R2-wt

NC mimics + PIK3R2-mut

miR-126-mimics + PIK3R2 -mut

**8.4 Experimental Step**

Twenty-four hours prior to transfection, 293T cells were seeded into a 12-well plate at a density of 2*105 cells per well. After 24 hours, when the cell density reached 70%-80%, the cells were divided into groups based on miR-126, LncRNA HOTAIR, and PIK3R2 and treated with the corresponding drugs. Each group was set up in triplicate.For transfection, the culture medium was replaced with serum-free DMEM, and after 6 hours, it was changed to 10% FBS DMEM without penicillin-streptomycin.The cells were then cultured for an additional 48 hours.Afterward, the cells were removed from the 12-well plate, the culture medium was aspirated, and the cells were washed 2-3 times with PBS. Subsequently, 300μL of cell lysis buffer was added, and the cells were incubated on ice for 10 minutes to ensure complete cell lysis. The lysate was then centrifuged at 4°C, 12000×g for 10 minutes, and the supernatant was collected.Luciferase and Renilla luciferase reaction solutions were prepared and equilibrated to room temperature. Fifty microliters of the above-mentioned cell lysate were transferred to a 96-well black fluorescence detection plate. Luciferase reaction solution was added, and the plate was shaken to mix well, followed by the measurement of firefly luciferase enzyme activity at a wavelength of 560 nm. Subsequently, Renilla luciferase reaction solution was added, and the plate was shaken to mix well, followed by the measurement of Renilla luciferase enzyme activity at a wavelength of 465 nm.

**8.5 The main reagents of the experiment**

| **Reagent** | **Manufacturer** | **Item number** |
| --- | --- | --- |
| DMEM high sugar medium | Solarbio | 12100 |
| GP-transfect-Mate | Gemma | G04009 |
| Double luciferase reporter gene detection kit | Beyotime | RG027 |

**8.6 Experimental equipment**

| **Instrument / equipment name** | **Brand / model** |
| --- | --- |
| Tabletop freezing centrifuge | Cence/H1650R |
| Analytical balance | Haimen Qilin Medical instrument Factory/TS-3D |
| Normal temperature centrifuge | Cence-H1650-W |
| Constant temperature incubator | Shanghai Zhicheng Analytical instrument/ZXGP-A2160 |
| Cell incubator | Thremo/3111 |
| Enzyme labeling instrument | Thremo/varioskan flash |
| Super clean station | Suzhou purification/SW-CJ-2D |

**9.Experimental report of immunofluorescence**

**9.1 Experimental Step**

With caution, PBS is removed, and 200μL of Saponin is added to the cell culture plate, allowing it to incubate at room temperature for 15 minutes. The cell culture plate is then placed on a shaker, and it is washed three times with PBS, each time for 5 minutes. Subsequently, 200μL of 3% BSA is evenly applied to cover the cells in the cell culture plate, and it is incubated at room temperature for 20 minutes. The sealing solution is gently aspirated, and 200μL of primary antibodies (AKT, p-AKT, VEGF, FGF2 at a 1:200 dilution, and PI3K at a 1:100 dilution in PBS) is added to the cell culture plate. The cell culture plate is placed flat inside a humid chamber and left to incubate overnight at 4°C.The cell culture plate is positioned on a shaker and washed three times for 5 minutes each with PBS. After a gentle air-drying, 200μL of secondary antibodies corresponding to the species of the primary antibodies (1:500) is added to the cell culture plate. Incubation is carried out at 37°C in the dark for 30 minutes.Following this, the cell culture plate is placed on a shaker and washed three times for 5 minutes each with PBS. After a brief air-drying, 200μL of DAPI staining solution is added to the cell culture plate, and it is incubated at room temperature for 5 minutes in the dark.The cell culture plate is then placed on a shaker and washed three times for 5 minutes each. After a slight air-drying, the coverslips are sealed with an anti-fade mounting medium for fluorescence.The slides are observed and images are captured using a fluorescence microscope. (DAPI with an ultraviolet excitation wavelength of 330-380nm and emission wavelength of 420nm emitting blue light; CY3 with a red light excitation wavelength of 510-560nm and emission wavelength of 590nm emitting red light).

**9.2The main reagents of the experiment**

| **Reagent** | **Manufacturer** | **Item number** |
| --- | --- | --- |
| 10×PBS | Servicebio | G4207-500ML |
| DAPI | Beyotime | C1002 |
| Anti-fluorescence quenching sealing solution | Beyotime | P0126 |
| BSA | Solarbio | A8020 |
| Cy3 labeled goat anti rabbit IgG (H+L) | Beyotime | A0516 |
| Cy3 labeled goat anti mouse IgG (H+L) | Beyotime | A0521 |
| Saponin | Beyotime | P0095-100ML |
| AKT | CST | 9272S |
| VEGF | Affinity | AF5131 |
| PI3K | Abcam | ab86714 |
| P-AKT | Affinity | AF0016 |
| FGF2 | Affinity | DF6038 |

**9.3Experimental equipment**

| **Name** | **Manufacturer** | **Model** |
| --- | --- | --- |
| Micro pipette | Thermo |  |
| low speed mini  centrifuge | Haimen Qilin Bell Instrument | LX 300 |
| positive fluorescence microscope | EVIDENT | CX43 |
| imaging system | EVIDENT | LIOO XC20 |
| scroll mixer | Hangzhou Qiwei Instrument | XW- 18DL |
| shaker | Haimen Qilin Bell Instrument | TS- 1000 |
| electrothermal constant temperature flume | Shanghai Sanfa Scientific Instrument | DK-420 |

**9.4 Result interpretation**

The nuclei stained by DAPI were blue under UV excitation, and the positive expression was red light labeled with corresponding fluorescein.

**10.Western blot experiment report**

**10.1 Experimental Step**

Cells were collected and 100μL of RIPA cell lysis buffer (containing 1mM PMSF) was added to each well of a 6-well plate. The cells were then placed on ice for 30 minutes. Afterward, the lysate was centrifuged at 12,000 rpm for 15 minutes to collect the supernatant, which contains total cellular proteins. The supernatant was mixed with 5X SDS-PAGE protein loading buffer at a 1:4 ratio and heated for 10 minutes to denature the proteins. After cooling, the protein samples were loaded into the wells of an SDS-PAGE gel, with each well receiving 30μg of sample.Pre-cut filter paper and a PVDF membrane, previously soaked in methanol for 3 minutes, were immersed in transfer buffer for 5 minutes. An electric current of 300 mA was applied for membrane transfer onto the PVDF membrane. After the transfer was complete, the protein membrane was immediately placed into a pre-prepared Western washing solution and washed for 5 minutes to remove any residual transfer buffer.The membrane was then blocked with a Western blocking solution (5% skim milk, or 5% BSA for phosphorylated proteins) and gently shaken on a shaker at room temperature for 2 hours. Primary antibodies, including anti-VEGF (1:1000, Affinity), anti-bFGF (1:500, Affinity), anti-PI3K (1:500, Affinity), anti-AKT (1:500, Affinity), and anti-p-AKT (1:500, Affinity), were used. The membrane was washed three times with PBST, and then incubated at room temperature with a secondary antibody conjugated with horseradish peroxidase (HRP) for 2 hours.The membrane was coated with an ECL solution, and exposure conditions were adjusted based on the varying levels of chemiluminescence. The bands on the membrane were analyzed using the "Image J" software.

**10.2The main reagents of the experiment**

| **Reagent** | **Manufacturer** | **Item number** |  |
| --- | --- | --- | --- |
| RIPA lysate (strong) | | Biosharp | BL504A |
| PMSF | | Biosharp | BL507A |
| SDS | | Solarbio | S8010 |
| Glycine | | Solarbio | G8200 |
| PAGE gel coagulant | | Solarbio | T8090 |
| Tris | | Solarbio | T8060 |
| Tween-20 | | Solarbio | T8220 |
| PVDF膜 | | Millipore | IPVH00010 |
| APS | | BBI Life Sciences | AB0072 |
| Acrylamide | | BBI Life Sciences | A1032 |
| Bis-Acrylamide | | Solarbio | M8200 |
| Methanol | | Shanghai Suyi | 无 |
| Anhydrous ethanol | | Shanghai Suyi | 无 |
| PBS buffer powder | | Zs-BIO | 无 |
| Pre stained  protein marker | | Thermo | 26616 |
| Western first and second antibody removal solution | | Beyotime | P0025 |
| ECL hypersensitive luminescence kit | | Thermo | 34094 |

**(1) SDS-PAGE gel preparation：**

The formulations of concentrated glue and separation glue are as follows:

5% concentrated adhesive（2ml） 10% separation adhesive（5ml）

Double distilled water 1.4ml Double distilled water 1.9ml

30% acrylamide mixture 330ul 30% acrylamide mixture 1.7ml

1.0molTris(PH6.8) 250ul 1.5molTris(PH8.8) 1.3ml

10%SDS 20ul 10%SDS 50ul

10%AP 20ul 10%AP 50ul

TEMED 2ul TEMED 2ul

**(2)Refer to the instruction manual of the first antibody**

| **Reagent** | **Manufacturer** | | **Item number** | **Molecular**  **weight** | **antibody source** | **dilution ratio** | **separation gel concentration** |
| --- | --- | --- | --- | --- | --- | --- | --- |
| β-actin | | Zs-BIO | TA-09 | 42kDa | Goat anti-mouse | 1:1000 |  |
| Goat anti-mouse IgG | | Zs-BIO | ZB-2305 |  |  | 1:10000 |  |
| Goat anti-rabbit IgG | | Zs-BIO | ZB-2301 |  |  | 1:10000 |  |
| AKT | | Affinity | AF6261 | 56kDa | Goat anti-rabbit | 1:500 | 10% |
| P-AKT | | Affinity | AF0016 | 56kDa | Goat anti-rabbit | 1:500 | 10% |
| PI3K | | Affinity | AF6241 | 84kDa | Goat anti-rabbit | 1:500 | 10% |
| VEGF | | Affinity | AF5131 | 27kDa | Goat anti-rabbit | 1:1000 | 10% |
| bFGF | | Affinity | DF6038 | 31kDa | Goat anti-rabbit | 1:500 | 10% |

**(3)Appendix: reagent formula**

**1. 10％SDS**

SDS                   10g

Distilled water is dissolved in 100ml

a water bath at 50 ℃ and stored at room temperature.

**2. 10%AP**

APS             0.1g

After dissolving in ultra-pure water, 1.0ml

it was stored at 4 ℃ for 1 week.

**3. 30% (w/v) acrylamide solution**

Acrylamide                29g

Bis-Acrylamide          1g

Add deionized water and fix the volume to 100ml to adjust the pH value of the solution to no more than 7.0. store it in a brown bottle at 4 ℃.

**4. 5×Electrophoretic solution formula(1000ml)**

Tris 15.1g

Glycine 94g

SDS 5.0g

Add deionized water and fix the volume to 1000ml

**5.Formula of transmembrane solution（1000ml）**

Tris 5.8g

Glycine 2.9g

SDS 0.37g

Methanol 200ml

Add deionized water and fix the volume to 1000ml

**6.1.0M Tris（pH 6.8）**

Tris 121.1g

Add about 800mL deionized water, add concentrated hydrochloric acid to adjust the pH value of the solution to 6.8, fix the volume to 1000mL, sterilize at high temperature and pressure and store at room temperature.

**7.1.5M Tris（pH 8.8）**

Tris 181.7g

Add about 800mL deionized water, add concentrated hydrochloric acid to adjust the pH value of the solution to 8.8, fix the volume to 1000mL, sterilize at high temperature and pressure and store at room temperature.

**10.3 Experimental equipment**

| **Name** | **Manufacturer** | **Model** |
| --- | --- | --- |
| Automatic ice maker | Changshu Xueke Electrical Appliances | IMS-20 |
| electrophoretic apparatus | Shanghai Tanon | EPS300 |
| electrophoretic trough | Shanghai Tanon | VE-180 |
| film transfer meter | Shanghai Tanon | VE-186 |
| room temperature microcentrifuge | Haimen Qilin Bell Instrument | LX 300 |
| high speed freezing centrifuge | Jiawen Instrument | JW-3021HR |
| pH meter | METTLER TOLEDO |  |
| horizontal shaker | Haimen Qilin Bell Instrument | TS-1000 |
| pure water machine | HHitech | Master-S30 UF |
| electrothermal constant temperature blast dryer | Sanfa Scientific Instrument | DHG-9070 |
| electronic balance | Jinghai Instrument | FA2004N |
| micropipette | Germany Eppendorf |  |
| magnetic heating agitator | Changzhou Renhe Instrument | JJ-79-1 |
| automatic exposure meter | Shanghai Peiqing Technology | JS-M6P |
|  |  |  |

**10.4 Result interpretation**

Analysis of Film Strip by ImageJ Software.

**11.Flow cytometry detection experiment**

**11.1 Experimental Step**

Cells were collected and centrifuged at 1000rpm for 5 minutes. After centrifugation, the cells were resuspended in 100μl of PBS. Subsequently, Anti-Human CD105, PE and FITC Anti-Human CD34 were added to the cells in respective volumes of 5μl each. The cells were then incubated for 20 minutes without exposure to light. Following the incubation, the cells were filtered and subjected to flow cytometry analysis using the Agilent NovoCyte flow cytometer. The light source utilized was an excitation light at 488 nm, and the filter settings were as follows: emission filter at 530/30 and 572/28. A minimum of 10,000 cells were collected at medium speed, and the light signals emitted by the cells were converted into electrical signals. The resulting data file was analyzed using the NovoExpress software .

**11.2The main reagents of the experiment**

| **Reagent** | **Manufacturer** | **Item number** |  | |
| --- | --- | --- | --- | --- |
| Trypsin | Beyotime | C0201 | |  |
| PBS | Servicebio | G4207 | |  |
| DMEM High glucose medium | Servicebio | G4512 | |  |
| Anti-Human CD105, PE | MULTI SCIENCES | 70-F1110502-25 | |  |
| FITC Anti-Human CD34 | Elabscience | E-AB-F1143C | |  |

**11.3Experimental equipment**

| **Name** | **Manufacturer** | **Model** |
| --- | --- | --- |
| Flow cytometry | Agilent | NovoCyte |
| Liquid transfer gun | Thermo scientific | FINNPIPETTE®F3 |
| Inverted microscope | OLYMPUS | CKX53 |
| Centrifuge machine | Anhui Jiawen instrument | JW-1024 |
| Incubator | Thermo scientific | 3111 |
| Super clean workbench | Shanghai Lichen instrument | SW-CJ-2D |
| Digital display water  bath | Shanghai Lichen Bangxi instrument | HH-4 |

**12.Rescue experiment**

In the PI3K/AKT activator group, Recilisib (MCE) was added. Other experimental reagents and equipment were the same as before.

Recilisib is a radioprotectant,can activate AKT, PI3K activities in cells.
